# Supplementary material for: Social isolation is linked to classical risk factors of Alzheimer’s disease-related dementias
Source: PLoS One. 2023 Feb 1;18(2):e0280471. doi: 10.1371/journal.pone.0280471 (PMC9891507; doi:10.1371/journal.pone.0280471)
Supplement: S1 File — (DOCX) [file pone.0280471.s001.docx]

**Supplementary Online Material**

**Supplementary Figure 1. Various ADRD-related lifestyle factors show strong association effects with loneliness and lack of social support across both cohorts**

| UKBB | 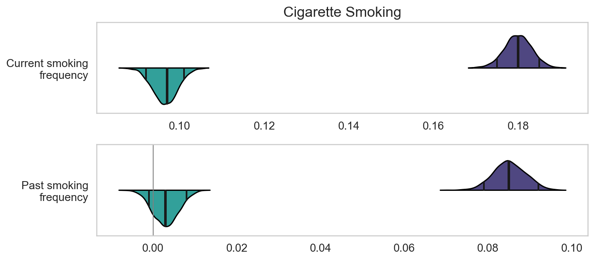  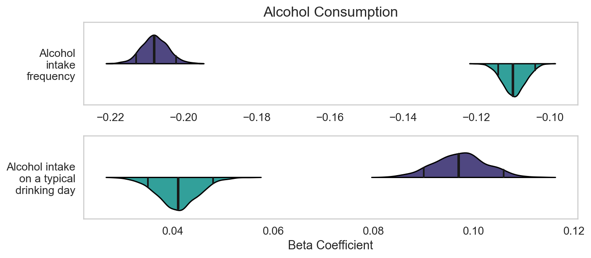  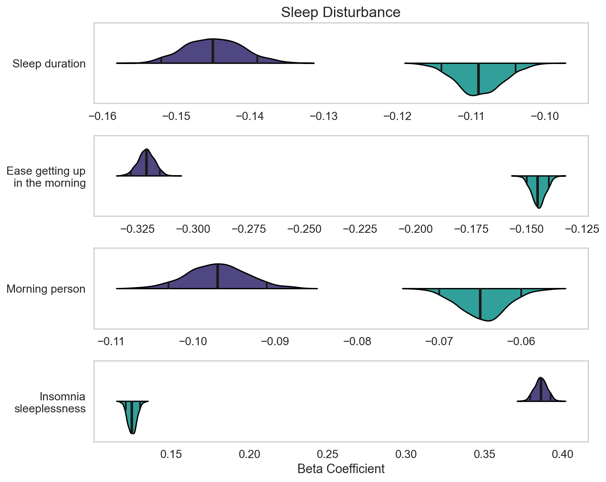  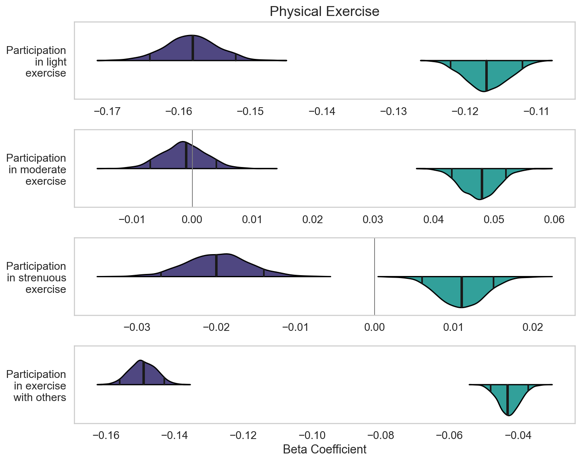  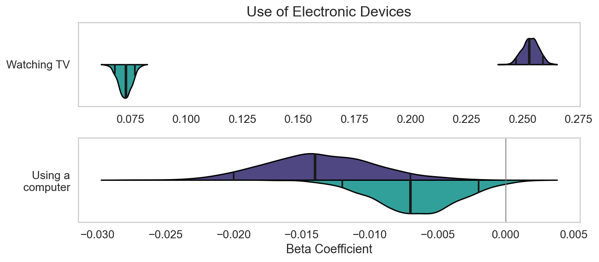  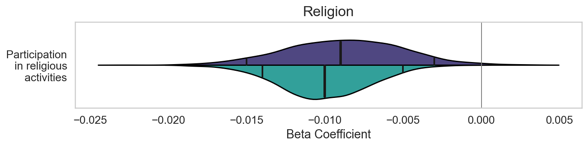 | CLSA | 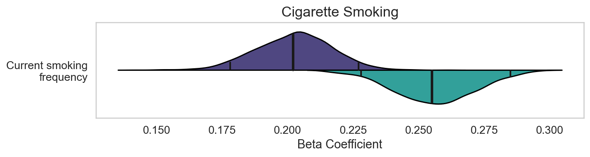  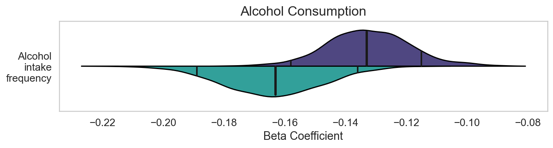  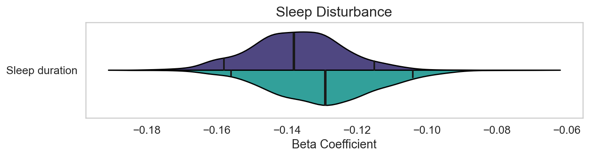  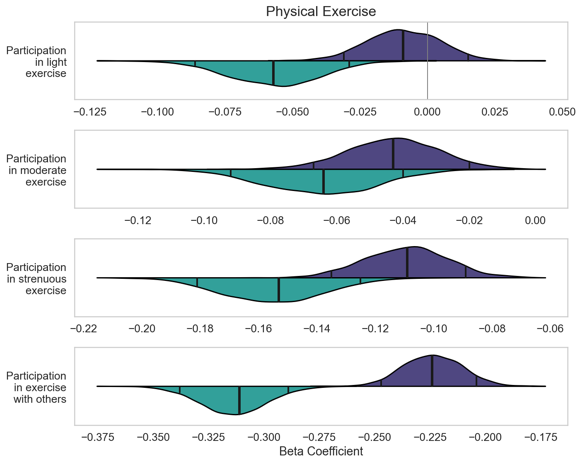  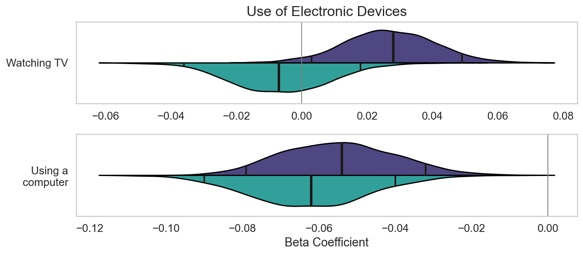  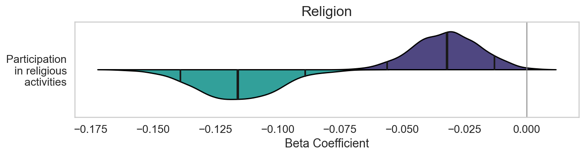  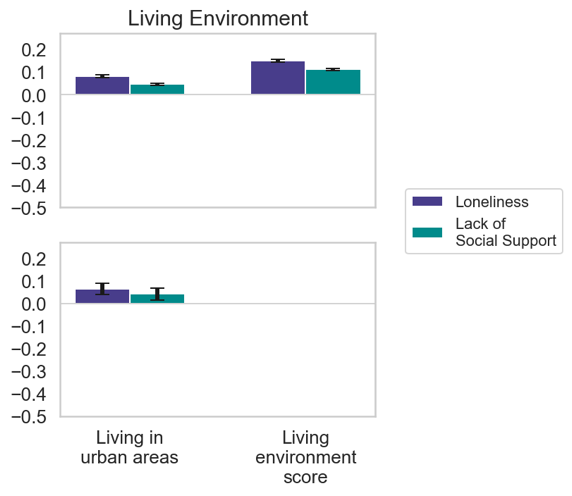 |
| --- | --- | --- | --- |

**Supplementary Figure 2. Physical health factors are related to social isolation.**

| UKBB | 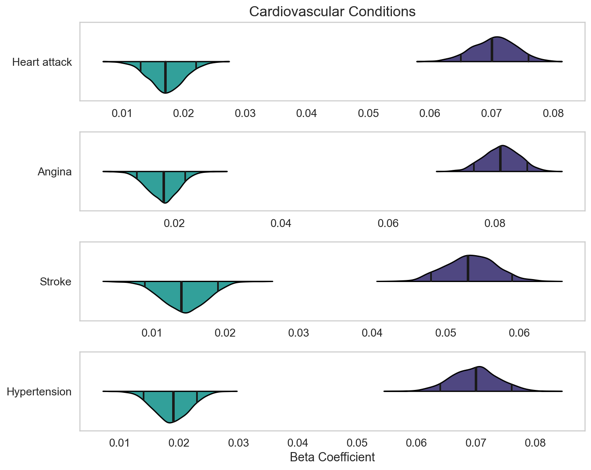  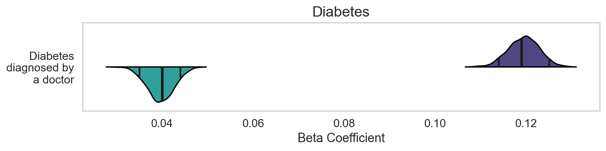  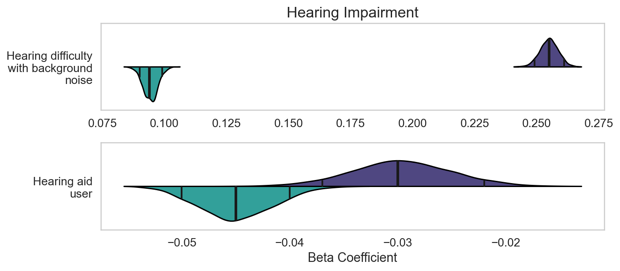  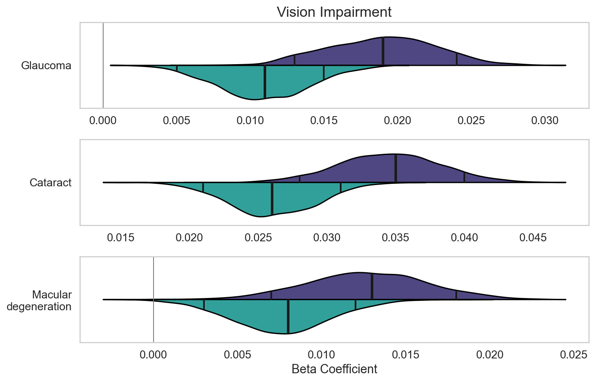 | CLSA | 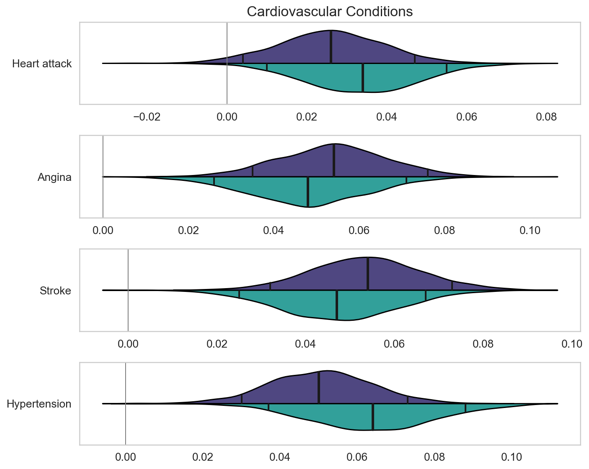  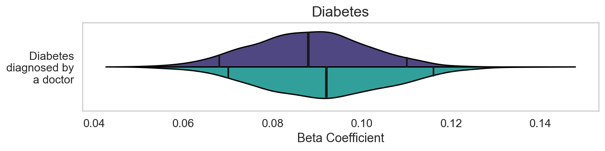  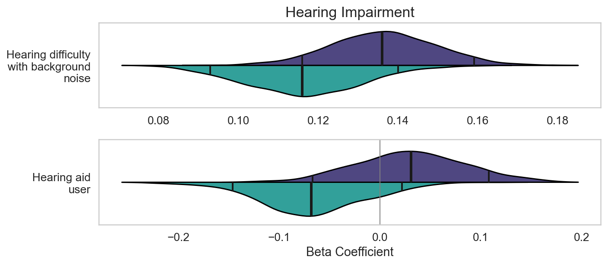  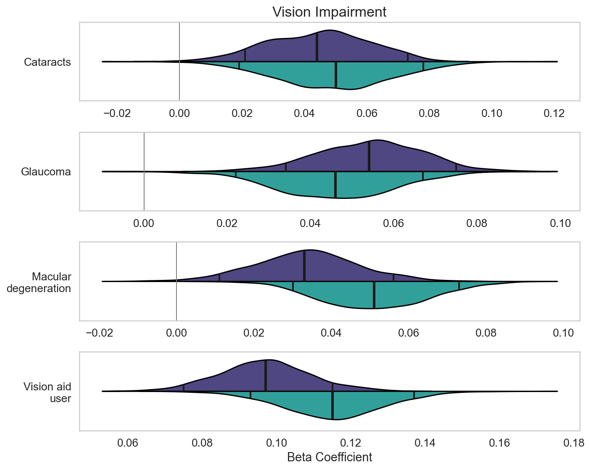  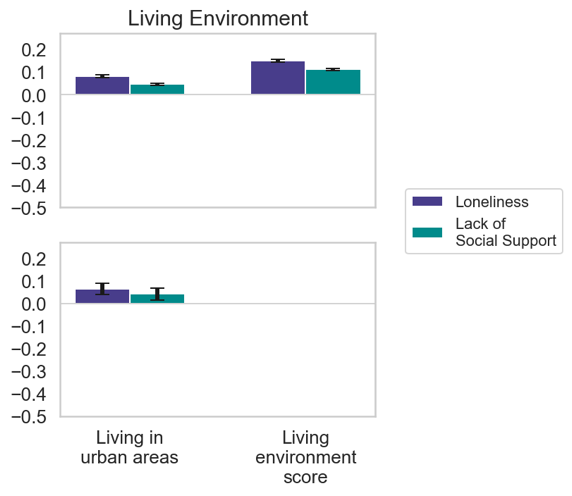 |
| --- | --- | --- | --- |
|  | | | |

**Supplementary Figure 3. Mental health factors show prominent association effects with social isolation.**

| UKBB | 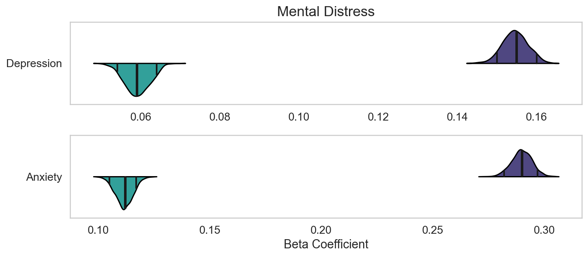  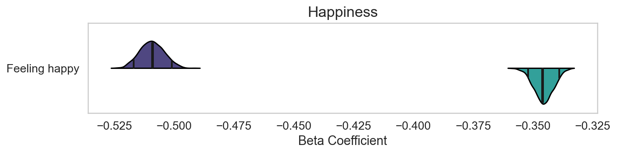  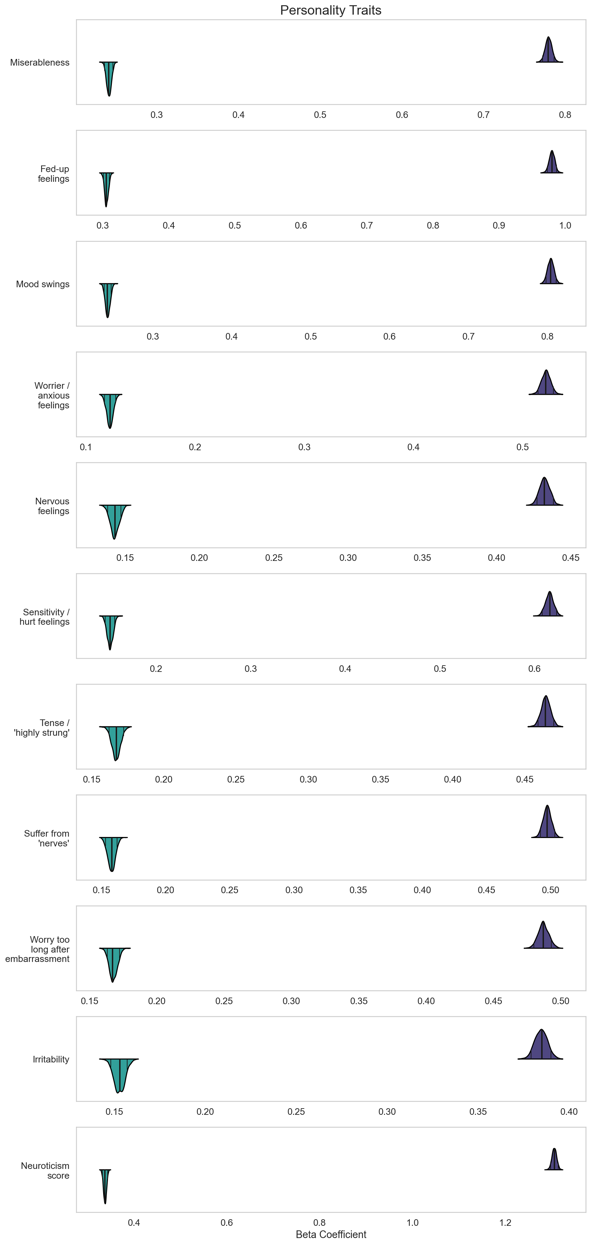 | CLSA | 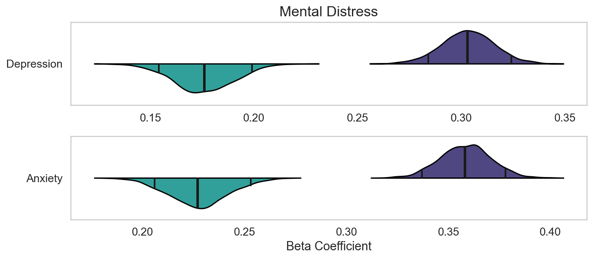  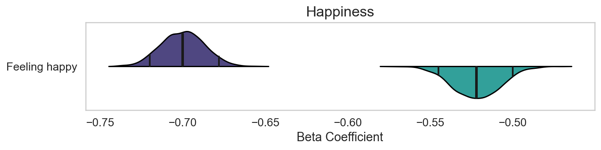  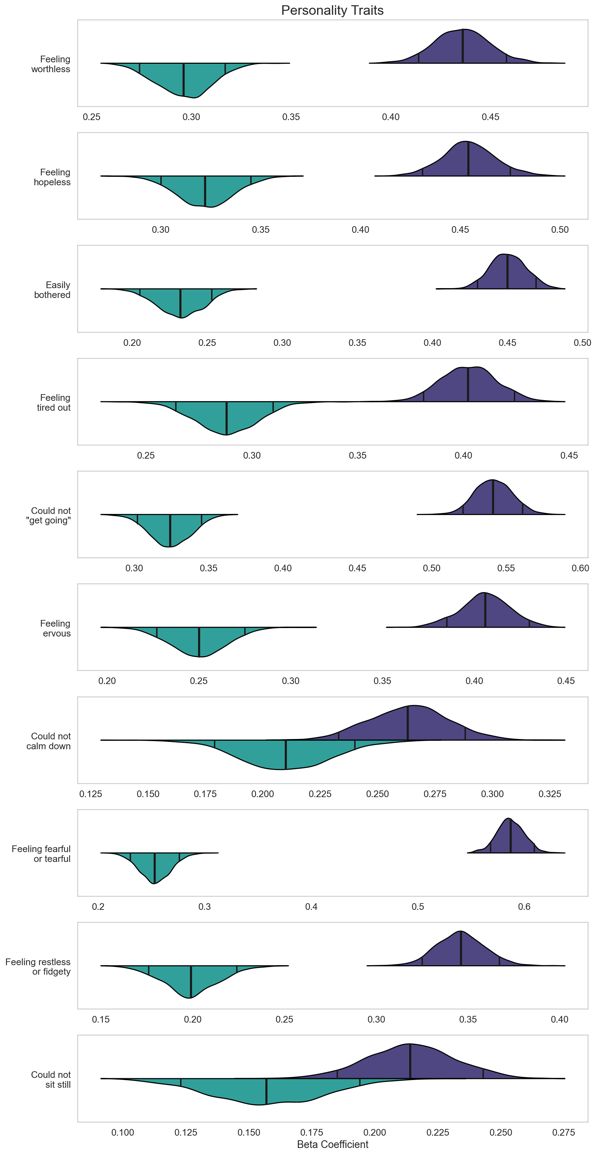  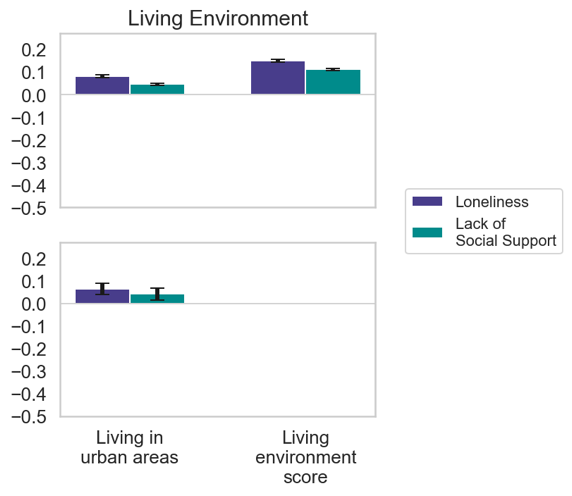 |
| --- | --- | --- | --- |

**Supplementary Figure 4. Societal risk factors exhibit salient association effects with social isolation.**

| UKBB | 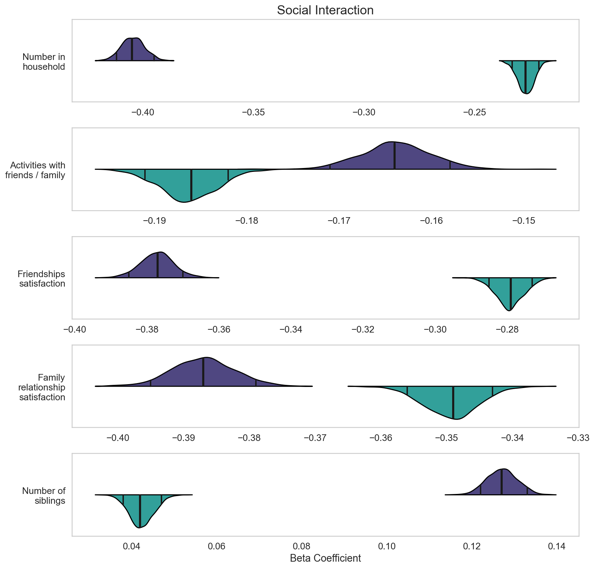  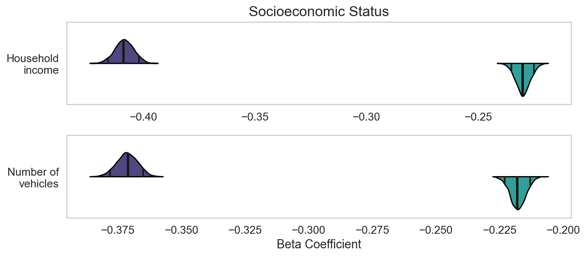  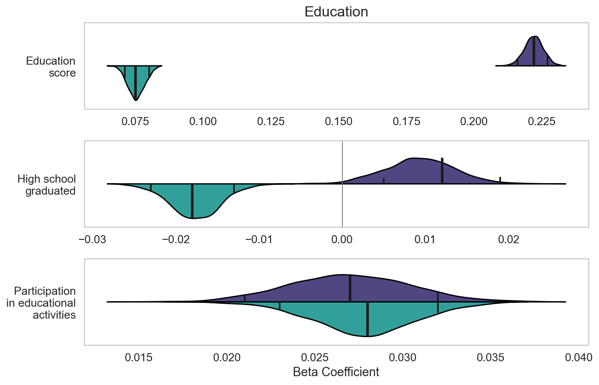  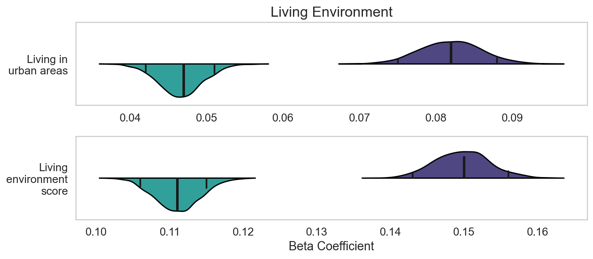 | CLSA | 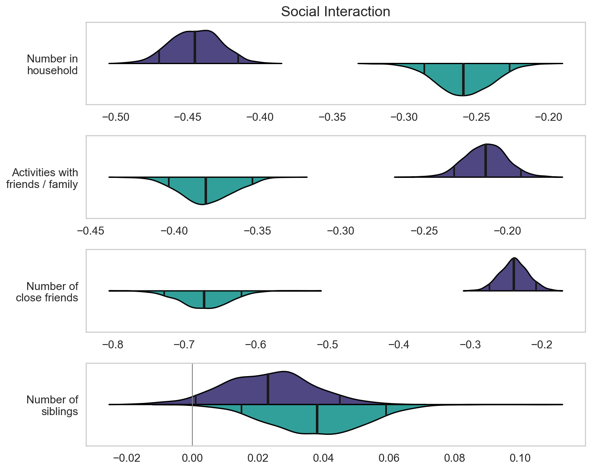  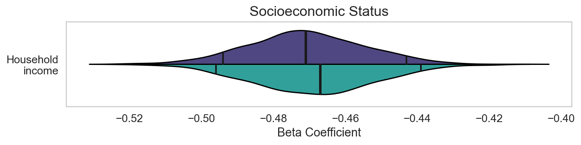  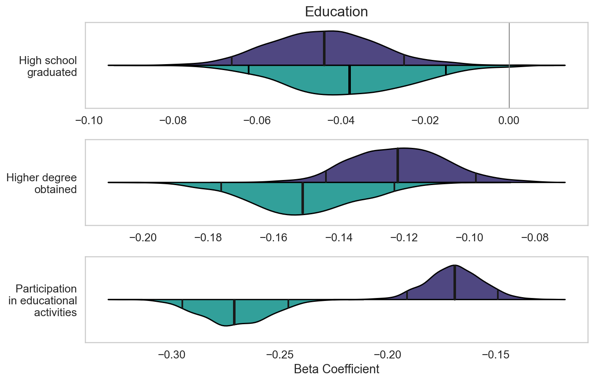  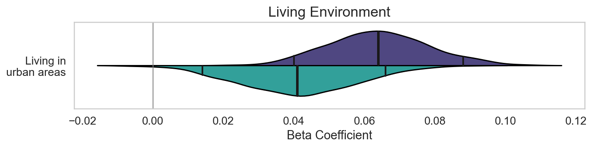  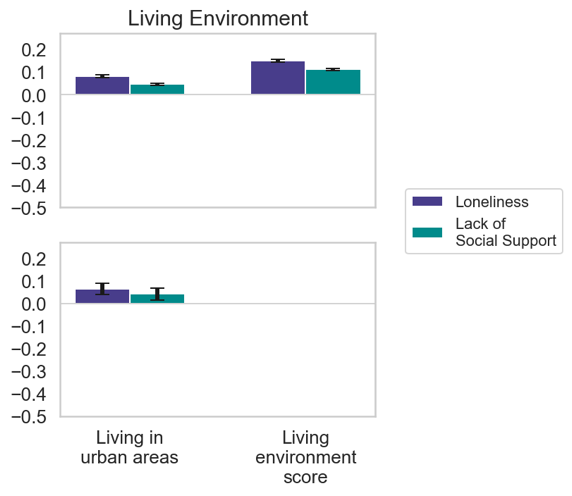 |
| --- | --- | --- | --- |
|  | | | |

**Supplementary Table 1. Details on the examined risk factors and social indicators per cohort.**

| **UK Biobank** | **Canadian Longitudinal Study on Aging** |
| --- | --- |
| **2020 - Loneliness, isolation**  "Do you often feel lonely?"   \| 0 \| No \| \| --- \| --- \| \| 1 \| Yes \| | **DEP_LONLY_COM - CES-D 10 scale: Frequency feel lonely**  “How often did you feel lonely?”   \| 1 \| All of the time (5-7 days) \| \| --- \| --- \| \| 2 \| Occasionally (3-4 days) \| \| 3 \| Some of the time (1-2 days) \| \| 4 \| Rarely or never (less than 1 day) \| \|  \|  \| |
| **2110 - Able to confide**  "How often are you able to confide in someone close to you?"   \| 0 \| Never or almost never \| \| --- \| --- \| \| 1 \| Once every few months \| \| 2 \| About once a month \| \| 3 \| About once a week \| \| 4 \| 2-4 times a week \| \| 5 \| Almost daily \| \|  \|  \| | **SSA_CONFID_COM - MOS scale: Support availability if need to confide**  “Someone to confide in or talk to about yourself or your problems?”   \| 1 \| None of the time \| \| --- \| --- \| \| 2 \| A little of the time \| \| 3 \| Some of the time \| \| 4 \| Most of the time \| \| 5 \| All of the time \| \|  \|  \| |
| **100347 - Current tobacco smoking**  "Do you smoke tobacco now?"   \| 0 \| No \| \| --- \| --- \| \| 1 \| Yes, on most or all days \| \| 2 \| Only occasionally \| | **SMK_CURRCG_COM - Current frequency of cigarettes smoked**  “At the present time, do you smoke cigarettes daily, occasionally or not at all?”   \| 1 \| Daily (at least one cigarette every day for the past 30 days) \| \| --- \| --- \| \| 2 \| Occasionally (at least one cigarette in the past 30 days, but not every day) \| \| 3 \| Not at all (you did not smoke at all in the past 30 days) \|   **.** |
| **100348 - Past tobacco smoking**  "In the past, how often have you smoked tobacco?"   \| 1 \| Smoked on most or all days \| \| --- \| --- \| \| 2 \| Smoked occasionally \| \| 3 \| Just tried once or twice \| \| 4 \| I have never smoked \| |  |
| **1558 - Alcohol intake frequency**  "About how often do you drink alcohol?"   \| 1 \| Daily or almost daily \| \| --- \| --- \| \| 2 \| Three or four times a week \| \| 3 \| Once or twice a week \| \| 4 \| One to three times a month \| \| 5 \| Special occasions only \| \| 6 \| Never \| | **ALC_FREQ_COM -** **Alcohol drinking frequency in past 12 months**  “About how often during the past 12 months did you drink alcohol?”   \| 1 \| Almost every day (incl. 6 times a week) \| \| --- \| --- \| \| 2 \| 4-5 times a week \| \| 3 \| 2-3 times a week \| \| 4 \| Once a week \| \| 5 \| 2-3 times a month \| \| 6 \| About once a month \| \| 7 \| Less than once a month \| \| 8 \| Never \| \|  \|  \| |
| **20403 - Amount of alcohol drunk on a typical drinking day**  "How many drinks* containing alcohol do you have on a typical day when you are drinking?"   \| 1 \| 1 or 2 \| \| --- \| --- \| \| 2 \| 3 or 4 \| \| 3 \| 5 or 6 \| \| 4 \| 7, 8, or 9 \| \| 5 \| 10 or more \| \|  \|  \| |  |
| **1160 - Sleep duration**  "About how many hours sleep do you get in every 24 hours? (please include naps)" | **SLE_HOUR_NB_COM - Number of sleep hours during past month**  Integer |
| **1200 - Sleeplessness / insomnia**  “Do you have trouble falling asleep at night or do you wake up in the middle of the night?"   \| 1 \| Never/rarely \| \| --- \| --- \| \| 2 \| Sometimes \| \| 3 \| Usually \| |  |
| **1170 - Getting up in morning**  "On an average day, how easy do you find getting up in the morning?"   \| 1 \| Not at all easy \| \| --- \| --- \| \| 2 \| Not very easy \| \| 3 \| Fairly easy \| \| 4 \| Very easy \| \|  \|  \| |  |
| **1180 - Morning/evening person (chronotype)**  "Do you consider yourself to be?"   \| 1 \| Definitely a ‘morning’ person \| \| --- \| --- \| \| 2 \| More a 'morning' than 'evening' person \| \| 3 \| More an 'evening' than a 'morning' person \| \| 4 \| Definitely an ‘evening’ person \| \|  \|  \| |  |
| **6164 - Types of physical activity in last 4 weeks**  "In the last 4 weeks did you spend any time doing the following? (You can select more than one answer)”   \| 1 \| Walking for pleasure (not as a means of transport) \| \| --- \| --- \| \| 2 \| Other exercises (eg: swimming, cycling, keep fit, bowling) \| \| 3 \| Strenuous sports \| \| 4 \| Light DIY (eg: pruning, watering the lawn) \| \| 5 \| Heavy DIY (eg: weeding, lawn mowing, carpentry, digging) \| | **PA2_LSPRT_MCQ - PASE scale: Frequency of participation in light sports**  “Over the past 7 days, how often did you engage in light sports or recreational activities such as bowling, golf with a cart, shuffleboard, badminton, fishing or other similar activities?”   \| 1 \| Never \| \| --- \| --- \| \| 2 \| Seldom (1 to 2 days) \| \| 3 \| Sometimes (3 to 4 days) \| \| 4 \| Often (5 to 7 days) \|   **PA2_MSPRT_MCQ - PASE scale: Frequency of participation in moderate sports**  “Over the past 7 days, how often did you engage in moderate sports or recreational activities such as ballroom dancing, hunting, skating, golf without a cart, softball or other similar activities?”   \| 1 \| Never \| \| --- \| --- \| \| 2 \| Seldom (1 to 2 days) \| \| 3 \| Sometimes (3 to 4 days) \| \| 4 \| Often (5 to 7 days) \|   **PA2_SSPRT_MCQ - PASE scale: Frequency of participation in strenuous sports**  “Over the past 7 days, how often did you engage in strenuous sports or recreational activities such as jogging, swimming, snowshoeing, cycling, aerobics, skiing, or other similar activities?”   \| 1 \| Never \| \| --- \| --- \| \| 2 \| Seldom (1 to 2 days) \| \| 3 \| Sometimes (3 to 4 days) \| \| 4 \| Often (5 to 7 days) \| \|  \|  \| |
| **6160 - Leisure/social activities**  "Which of the following do you attend once a week or more often? (You can select more than one)"   \| 1 \| Sports club or gym \| \| --- \| --- \| \| 2 \| Pub or social club \| \| 3 \| Religious group \| \| 4 \| Adult education class \| \| 5 \| Other group activity \| | **SPA_SPORT_COM - Frequency of participation in sports or physical activities with others**  “In the past 12 months, how often did you participate in Sports or physical activities that you do with other people?”   \| 1 \| At least once a day \| \| --- \| --- \| \| 2 \| At least once a week \| \| 3 \| At least once a month \| \| 4 \| At least once a year \| \| 5 \| Never \|   **SPA_CLUB_COM - Frequency of participation in clubs or fraternal organization activities**  “In the past 12 months, how often did you participate in Service club or fraternal organization activities?”   \| 1 \| At least once a day \| \| --- \| --- \| \| 2 \| At least once a week \| \| 3 \| At least once a month \| \| 4 \| At least once a year \| \| 5 \| Never \|   **SPA_CHRCH_COM - Frequency of participation in religious activities**  “In the past 12 months, how often did you participate in church or religious activities such as services, committees or choirs?”   \| 1 \| At least once a day \| \| --- \| --- \| \| 2 \| At least once a week \| \| 3 \| At least once a month \| \| 4 \| At least once a year \| \| 5 \| Never \|   **SPA_EDUC_COM - Frequency of participation in educational or cultural activities**  “In the past 12 months, how often did you participate in Educational and cultural activities involving other people such as attending courses, concerts, plays, or visiting museums?”   \| 1 \| At least once a day \| \| --- \| --- \| \| 2 \| At least once a week \| \| 3 \| At least once a month \| \| 4 \| At least once a year \| \| 5 \| Never \| \|  \|  \| |
| **1070 - Time spent watching television (TV)**  "In a typical DAY, how many hours do you spend watching TV? (Put 0 if you do not spend any time doing it)" | **PA2_SIT_TV_MCQ - PASE scale: Participated in sitting activities - Watching TV**   \| 0 \| No \| \| --- \| --- \| \| 1 \| Yes \| |
| **1080 - Time spent using computer**  "In a typical DAY, how many hours do you spend using the computer? (Do not include using a computer at work; put 0 if you do not spend any time doing it)" | **PA2_SIT_COM_MCQ - PASE scale: Participated in sitting activities - Computer activities**   \| 0 \| No \| \| --- \| --- \| \| 1 \| Yes \| |
|  | |
| **6150 - Vascular/heart problems diagnosed by doctor**  "Has a doctor ever told you that you have had any of the following conditions?”   \| 1 \| Heart attack \| \| --- \| --- \| \| 2 \| Angina \| \| 3 \| Stroke \| \| 4 \| High blood pressure \| | **CCC_AMI_COM - Heart attack or myocardial infarction**  “Has a doctor ever told you that you have had a heart attack or myocardial infarction?”   \| 1 \| Yes \| \| --- \| --- \| \| 2 \| No \|   **CCC_ANGI_COM - Angina (or chest pain due to heart disease)**  “Has a doctor ever told you that you have angina (or chest pain due to heart disease)?”   \| 1 \| Yes \| \| --- \| --- \| \| 2 \| No \|   **CCC_HBP_COM - High blood pressure or hypertension**  “Has a doctor ever told you that you have high blood pressure or hypertension?”   \| 1 \| Yes \| \| --- \| --- \| \| 2 \| No \|   **CCC_CVA_COM - Stroke or CVA (cerebrovascular accident)**  Has a doctor ever told you that you have experienced a Stroke or CVA (cerebrovascular accident)?   \| 1 \| Yes \| \| --- \| --- \| \| 2 \| No \| \|  \|  \| |
| **2443 - Diabetes diagnosed by doctor**  "Has a doctor ever told you that you have diabetes?"   \| 0 \| No \| \| --- \| --- \| \| 1 \| Yes \| | **DIA_DIAB_COM - Diabetes, borderline diabetes or high blood sugar**  “Has a doctor ever told you that you have diabetes, borderline diabetes or that your blood sugar is high?”   \| 1 \| Yes \| \| --- \| --- \| \| 2 \| No \| \|  \|  \| |
| **2257 - Hearing difficulty/problems with background noise**  "Do you find it difficult to follow a conversation if there is background noise (such as TV, radio, children playing)?"   \| 0 \| No \| \| --- \| --- \| \| 1 \| Yes \| | **HRG_NOIS_COM - Hearing difficulty with background noise**  “Do you find it difficult to follow a conversation if there is background noise, such as TV, radio or children playing, even if using a hearing aid as usual?”   \| 1 \| Yes \| \| --- \| --- \| \| 2 \| No \| |
| **3393 - Hearing aid user**  "Do you use a hearing aid most of the time?"   \| 0 \| No \| \| --- \| --- \| \| 1 \| Yes \| | **HRG_AID_COM - Uses any type of hearing aids**  “Do you use any aids, specialized equipment, or services for persons who are deaf or hard of hearing, for example, a volume control telephone or TV decoder?”   \| 1 \| Yes \| \| --- \| --- \| \| 2 \| No \| \|  \|  \| |
| **6148 - Eye problems/disorders**  "Has a doctor told you that you have any of the following problems with your eyes?”   \| 1 \| Diabetes related eye disease \| \| --- \| --- \| \| 2 \| Glaucoma \| \| 3 \| Injury or trauma resulting in loss of vision \| \| 4 \| Cataract \| \| 5 \| Macular degeneration \| \| 6 \| Other serious eye condition \| | **ICQ_CATRCT_COM – Ever had cataracts**  “Has a doctor ever told you that you have cataracts?”   \| 1 \| Yes \| \| --- \| --- \| \| 2 \| No \|   **ICQ_GLAUC_COM – Ever had glaucoma**  “Has a doctor ever told you that you have glaucoma?”   \| 1 \| Yes \| \| --- \| --- \| \| 2 \| No \|   **CCC_MACDEG_COM - Macular degeneration**  “Has a doctor ever told you that you have macular degeneration?”   \| 1 \| Yes \| \| --- \| --- \| \| 2 \| No \| \|  \|  \| |
|  | **VIS_AID_COM - Vision aids use**  “Besides glasses or contact lenses, do you use any aids or specialized equipment for persons who are blind or visually impaired, for example, magnifiers or Braille reading materials?”   \| 1 \| Yes \| \| --- \| --- \| \| 2 \| No \| \|  \|  \| |
|  | |
| **20544 - Mental health problems ever diagnosed by a professional**  "Have you been diagnosed with one or more of the following mental health problems by a professional, even if you don't have it currently?”   \| 1 \| Social anxiety or social phobia \| \| --- \| --- \| \| 2 \| Schizophrenia \| \| 3 \| Any other type of psychosis or psychotic illness \| \| 4 \| A personality disorder \| \| 5 \| Any other phobia (eg disabling fear of heights or spiders) \| \| 6 \| Panic attacks \| \| 7 \| Obsessive compulsive disorder (OCD) \| \| 10 \| Mania, hypomania, bipolar or manic-depression \| \| **11** \| **Depression** \| \| 12 \| Bulimia nervosa \| \| 13 \| Psychological over-eating or binge-eating \| \| 14 \| Autism, Asperger’s or autistic spectrum disorder \| \| 15 \| Anxiety, nerves or generalized anxiety disorder \| \| 16 \| Anorexia nervosa \| \| 17 \| Agoraphobia \| \| 18 \| Attention deficit or attention deficit and hyperactivity disorder (ADD/ADHD) \| \|  \|  \| | **DPR_CLINDEP_COM - Clinical depression**   \| 1 \| Yes \| \| --- \| --- \| \| 2 \| No \| |
| **20506 - Recent feelings of nervousness or anxiety**  "Over the last 2 weeks, how often have you been bothered by any of the following problems? [anxiety symptoms] Feeling nervous, anxious or on edge"   \| 1 \| Not at all \| \| --- \| --- \| \| 2 \| Several days \| \| 3 \| More than half the days \| \| 4 \| Nearly every day \|   **.** | **PER_ANX_MCQ - TIPI scale: Sees oneself as anxious and easily upset**  “Has a doctor ever told you that you have an anxiety disorder such as a phobia, obsessive-compulsive disorder or a panic disorder?”   \| 1 \| Yes \| \| --- \| --- \| \| 2 \| No \| |
| **20127 - Neuroticism score**  This is an externally derived summary score of neuroticism, based on 12 neurotic behaviour domains. Questions included:   - Does your mood often go up and down? - Do you ever feel 'just miserable' for no reason? - Are you an irritable person? - Are your feelings easily hurt? - Do you often feel 'fed-up'? - Would you call yourself a nervous person? - Are you a worrier? - Would you call yourself tense or 'highly strung'? - Do you worry too long after an embarrassing experience? - Do you suffer from 'nerves'? - Do you often feel lonely? - Are you often troubled by feelings of guilt?   Participants could answer Yes, No, Do not know or Prefer not to answer.  This field summarises the number of Yes answers across these twelve questions into a single integer score for each participant. |  |
| **1920 - Mood swings**  "Does your mood often go up and down?"   \| 0 \| No \| \| --- \| --- \| \| 1 \| Yes \|   **1930 - Miserableness**  "Do you ever feel 'just miserable' for no reason?"   \| 0 \| No \| \| --- \| --- \| \| 1 \| Yes \|   **1940 - Irritability**  "Are you an irritable person?"   \| 0 \| No \| \| --- \| --- \| \| 1 \| Yes \|   **1950 Sensitivity / hurt feelings**  "Are your feelings easily hurt?"   \| 0 \| No \| \| --- \| --- \| \| 1 \| Yes \|   **1960 - Fed-up feelings**  "Do you often feel 'fed-up'?"   \| 0 \| No \| \| --- \| --- \| \| 1 \| Yes \|   **1970 - Nervous feelings**  "Would you call yourself a nervous person?"   \| 0 \| No \| \| --- \| --- \| \| 1 \| Yes \|   **1980 - Worrier / anxious feelings**  "Are you a worrier?"   \| 0 \| No \| \| --- \| --- \| \| 1 \| Yes \|   **1990 - Tense / 'highly strung'**  "Would you call yourself tense or 'highly strung'?"   \| 0 \| No \| \| --- \| --- \| \| 1 \| Yes \|   **2000 - Worry too long after embarrassment**  "Do you worry too long after an embarrassing experience?"   \| 0 \| No \| \| --- \| --- \| \| 1 \| Yes \|   **2010 - Suffer from 'nerves'**  "Do you suffer from 'nerves'?"   \| 0 \| No \| \| --- \| --- \| \| 1 \| Yes \| \|  \|  \| | **K10_WRTHLSS_MCQ - K10 scale: Frequency feeling worthless - past 30 days**   \| 1 \| All of the time \| \| --- \| --- \| \| 2 \| Most of the time \| \| 3 \| Some of the time \| \| 4 \| A little of the time \| \| 5 \| None of the time \|   **K10_HPLS_MCQ - K10 scale: Frequency hopeless - past 30 days**   \| 1 \| All of the time \| \| --- \| --- \| \| 2 \| Most of the time \| \| 3 \| Some of the time \| \| 4 \| A little of the time \| \| 5 \| None of the time \|   **K10_TIRED_MCQ - K10 scale: Frequency tired out - past 30 days**   \| 1 \| All of the time \| \| --- \| --- \| \| 2 \| Most of the time \| \| 3 \| Some of the time \| \| 4 \| A little of the time \| \| 5 \| None of the time \|   **K10_NRVS_MCQ - K10 scale: Frequency nervous - past 30 days**   \| 1 \| All of the time \| \| --- \| --- \| \| 2 \| Most of the time \| \| 3 \| Some of the time \| \| 4 \| A little of the time \| \| 5 \| None of the time \|   **K10_NRVSCLMD_MCQ - K10 scale: Frequency could not calm down - past 30 days**   \| 1 \| All of the time \| \| --- \| --- \| \| 2 \| Most of the time \| \| 3 \| Some of the time \| \| 4 \| A little of the time \| \| 5 \| None of the time \|   **K10_RSTLS_MCQ - K10 scale: Frequency restless or fidgety - past 30 days**   \| 1 \| All of the time \| \| --- \| --- \| \| 2 \| Most of the time \| \| 3 \| Some of the time \| \| 4 \| A little of the time \| \| 5 \| None of the time \|   **K10_RSTLSSTL_MCQ - K10 scale: Frequency can not sit still - past 30 days**   \| 1 \| All of the time \| \| --- \| --- \| \| 2 \| Most of the time \| \| 3 \| Some of the time \| \| 4 \| A little of the time \| \| 5 \| None of the time \|   **DEP_FRFL_COM - CES-D 10 scale: Frequency feel fearful or tearful**  “How often did you feel fearful or tearful?”   \| 1 \| All of the time (5-7 days) \| \| --- \| --- \| \| 2 \| Occasionally (3-4 days) \| \| 3 \| Some of the time (1-2 days) \| \| 4 \| Rarely or never (less than 1 day) \|   **DEP_BOTR_COM - CES-D 10 scale: Frequency easily bothered**  “How often were you bothered by things that usually don’t bother you?”   \| 1 \| All of the time (5-7 days) \| \| --- \| --- \| \| 2 \| Occasionally (3-4 days) \| \| 3 \| Some of the time (1-2 days) \| \| 4 \| Rarely or never (less than 1 day) \|   **DEP_GTGO_COM - CES-D 10 scale: Frequency feel could not 'get going'**  “How often did you feel that you could not “get going”?”   \| 1 \| All of the time (5-7 days) \| \| --- \| --- \| \| 2 \| Occasionally (3-4 days) \| \| 3 \| Some of the time (1-2 days) \| \| 4 \| Rarely or never (less than 1 day) \| \|  \|  \| |
| **4526 - Happiness**  "In general how happy are you?"   \| 1 \| Extremely happy \| \| --- \| --- \| \| 2 \| Very happy \| \| 3 \| Moderately happy \| \| 4 \| Moderately unhappy \| \| 5 \| Very unhappy \| \| 6 \| Extremely unhappy \|   **.** | **DEP_HAPP_COM - CES-D 10 scale: Frequency feel happy**  "How often were you happy?”   \| 1 \| All of the time (5-7 days) \| \| --- \| --- \| \| 2 \| Occasionally (3-4 days) \| \| 3 \| Some of the time (1-2 days) \| \| 4 \| Rarely or never (less than 1 day) \| \|  \|  \| |
|  | |
| **709 - Number in household**  "Including yourself, how many people are living together in your household? (Include those who usually live in the house such as students living away from home during term, partners in the armed forces or professions such as pilots)" | **SN_LIVH_NB_COM - Number of people living in household (excluding the participant)**  “How many people, not including yourself, currently live in your household?” |
| **1031 - Frequency of friend/family visits**  "How often do you visit friends or family or have them visit you?"   \| 1 \| Almost daily \| \| --- \| --- \| \| 2 \| 2-4 times a week \| \| 3 \| About once a week \| \| 4 \| About once a month \| \| 5 \| Once every few months \| \| 6 \| Never or almost never \| \| 7 \| No friends/family outside household \| \|  \|  \| | **SPA_OUTS_COM - Frequency of participation in family / friends activities out of household**  “In the past 12 months, how often did you participate in family- or friendship-based activities outside the household?”   \| 1 \| At least once a day \| \| --- \| --- \| \| 2 \| At least once a week \| \| 3 \| At least once a month \| \| 4 \| At least once a year \| \| 5 \| Never \| |
| **4570 - Friendships satisfaction**  "In general, how satisfied are you with your friendships?"   \| 1 \| Extremely happy \| \| --- \| --- \| \| 2 \| Very happy \| \| 3 \| Moderately happy \| \| 4 \| Moderately unhappy \| \| 5 \| Very unhappy \| \| 6 \| Extremely unhappy \|   **.** |  |
| **4559 - Family relationship satisfaction**  "In general, how satisfied are you with your family relationships?"   \| 1 \| Extremely happy \| \| --- \| --- \| \| 2 \| Very happy \| \| 3 \| Moderately happy \| \| 4 \| Moderately unhappy \| \| 5 \| Very unhappy \| \| 6 \| Extremely unhappy \|   **.** |  |
|  | **SN_FRND_NB_COM - Number of close friends**  **“**Not counting family members, how many people do you consider close friends – that is, people you can confide in and talk over personal matters with?” |
| **1873 - Number of full brothers**  "How many brothers do you have? (Please include those who have died, and twin brothers. Do not include half-brothers, step-brothers or adopted brothers)"  **1883 - Number of full sisters**  "How many sisters do you have? (Please include those who have died, and twin sisters. Do not include half-sisters, step-sisters or adopted sisters)" | **SN_SIBLIV_NB_COM - Number of living siblings**  “How many, if any, living siblings (sisters, brothers) do you have?” |
| **728 - Number of vehicles in household**  "How many cars or vans are owned, or available for use, by you or members of your household? (Please include company vehicles if available for private use)"   \| 1 \| None \| \| --- \| --- \| \| 2 \| One \| \| 3 \| Two \| \| 4 \| Three \| \| 5 \| Four or more \| \|  \|  \| |  |
| **738 - Average total household income before tax**  "What is the average total income before tax received by your household?"   \| 1 \| Less than 18,000 \| \| --- \| --- \| \| 2 \| 18,000 to 30,999 \| \| 3 \| 31,000 to 51,999 \| \| 4 \| 52,000 to 100,000 \| \| 5 \| Greater than 100,000 \| | **INC_TOT_COM - Total household income**  “What is your best estimate of the total household income received by all household members, from all sources, before taxes and deductions, in the past 12 months?”   \| 1 \| Less than $20,000 \| \| --- \| --- \| \| 2 \| $20,000 or more, but less than $50,000 \| \| 3 \| $50,000 or more, but less than $100,000 \| \| 4 \| $100,000 or more, but less than $150,000 \| \| 5 \| $150,000 or more \| \|  \|  \| |
| **26414 - Education score (England)**  This domain measures the extent of deprivation in terms of education, skills and training in an area. The indicators are structured into two sub-domains: one relating to children and young people and one relating to adult skills. These two sub-domains are designed to reflect the 'flow' and 'stock' of educational disadvantage within an area respectively. |  |
| **845 - Age completed full time education**  "At what age did you complete your continuous full-time education?" | **ED_HSGR_COM - Education high school graduated**  “Did you graduate from high school (secondary school)?”   \| 1 \| Yes \| \| --- \| --- \| \| 2 \| No \| \|  \|  \| |
|  | **ED_HIGH_COM – Education highest degree**  “What is the highest degree, certificate, or diploma you have obtained?”   \| 1 \| No post-secondary degree, certificate, or diploma \| \| --- \| --- \| \| 2 \| Trade certificate or diploma from a vocational school or apprenticeship training \| \| 3 \| Non-university certificate or diploma from a community college, CEGEP, school of nursing, etc. \| \| 4 \| University certificate below bachelor’s level \| \| 5 \| Bachelor’s degree \| \| 6 \| University degree or certificate above bachelor’s degree \| \|  \|  \| |
| **20118 - Home area population density - urban or rural**  The classification is derived by combining each participant’s home postcode with data generated from the 2001 census.   \| 1 \| England/Wales - Urban - sparse \| \| --- \| --- \| \| 2 \| England/Wales - Town and Fringe – sparse \| \| 3 \| England/Wales - Village – sparse \| \| 4 \| England/Wales - Hamlet and Isolated dwelling – sparse \| \| 5 \| England/Wales - Urban - less sparse \| \| 6 \| England/Wales - Town and Fringe - less sparse \| \| 7 \| England/Wales - Village - less sparse \| \| 8 \| England/Wales - Hamlet and Isolated Dwelling - less sparse \| \| 9 \| Postcode not linkable \| \| 11 \| Scotland - Large Urban Area \| \| 12 \| Scotland - Other Urban Area \| \| 13 \| Scotland - Accessible Small Town \| \| 14 \| Scotland - Remote Small Town \| \| 15 \| Scotland - Very Remote Small Town \| \| 16 \| Scotland - Accessible Rural \| \| 17 \| Scotland - Remote Rural \| \| 18 \| Scotland - Very Remote Rural \| \|  \|  \| | **SDC_URBAN_RURAL_COM – Urban / rural classification**   \| 0 \| Rural \| \| --- \| --- \| \| 1 \| Urban core \| \| 2 \| Urban fringe \| \| 3 \| Urban population centre outside CMA and CA \| \| 6 \| Secondary core \| \| 9 \| Postal code link to dissemination area \| |

*A "drink" is defined as one unit of alcohol.
